# Plasma Endothelin-1 Levels: Non-Predictors of Alzheimer’s Disease Reveal Age Correlation in African American Women

**DOI:** 10.3390/jcm14020635

**Published:** 2025-01-19

**Authors:** Irene A. Zagol-Ikapitte, Mohammad A. Tabatabai, Derek M. Wilus, Donald J. Alcendor

**Affiliations:** 1 Proteomics Laboratory, Mass Spectrometry Research Center, Vanderbilt University Medical Center, Nashville, TN 37232, USA; ikapitte@vanderbilt.edu; 2 School of Global Health, Meharry Medical College, Nashville, TN 37208, USA; 3 Center for AIDS Health Disparities Research, Department of Microbiology, Immunology, and Physiology, Meharry Medical College, School of Medicine, Nashville, TN 37208, USA

**Keywords:** endothelin-1, Alzheimer’s disease, biomarker, disparities, minorities

## Abstract

**Background/Objectives:** Alzheimer’s disease (AD) and related dementias (ADRD) disproportionately impact racial and ethnic minorities. Contributing biological factors that explain this disparity have been elusive. Moreover, non-invasive biomarkers for early detection of AD are needed. Endothelin-1 (ET-1), a vasoconstrictive factor linked to cerebral vascular disease pathology and neuronal injury, could provide insights to better understand racial disparities in AD. As a potent vasoconstrictive peptide that regulates contractions in smooth muscle, endothelial cells, and pericytes, ET-1 may result in cerebral vascular constriction, leading to cerebral hypoperfusion; over time, this may result in neuronal injury, contributing to the pathology of AD. The role of the ET-1 system as a driver of ethnic disparities in AD requires further investigation. In the United States (U.S.), ET-1 dysregulation in Hispanic/Latinx (H/L) ethnic populations has largely been unexplored. Genetics linking ET-1 dysregulation and racial disparities in AD also require further investigation. In this study, we examined the role of the ET-1 protein in human plasma as a potential biomarker with predictive value for correlating with the development of AD by age, race, and sex. **Methods:** We examined ET-1 protein levels using quantitative mass spectrometry in AA and NHW patients with AD, along with controls. **Results:** A partial correlation between age at draw and ET-1, stratified by race and sex, while controlling for AD status, was significant for female AAs (r = 0.385, *p* = 0.016). When the data were not stratified but controlled for AD status, the partial correlation between age at draw and ET-1 was not significant (r = 0.108, *p* = 0.259). **Conclusions:** Based on the small number of plasma specimens and no plasma specimens from H/L individuals with AD, we conclude that ET-1 was clearly not a significant factor in predicting AD in this study and will require a larger scale study for validation.

## 1. Introduction

Alzheimer’s disease (AD) is the most common form of dementia globally. By 2050, it is estimated that it will affect approximately 131.5 million people, with no curative interventions. AD is a complex multifactorial neurodegenerative disease with an assortment of pathological pathways, including synaptic dysfunctions and deficits in synaptic plasticity, resulting in progressive cognitive decline [1,2]. Multiple studies suggest that the proposed cause of AD could be linked to cholinergic deficiency [3], amyloid beta (Aβ) toxicity [4], tau protein hyperphosphorylation [5], synaptic dysfunction [6], oxidative stress [7], and neuroinflammation [8]. AD results in progressive cognitive decline and memory impairment, aggressive behavior, anxiety, depression, feelings of apathy, and sleep disorders [9]. Recognized pathophysiology of AD includes atrophy of the frontal, temporal, and parietal lobes, enlargement of the temporal horn of the lateral ventricle, and atrophy of the entorhinal cortex, amygdala, and hippocampus. Atrophic lesions are characterized as having accumulations of beta-amyloid deposition in neurons and glial cells with associated Tau protein aggregation [10,11]. There is also dysregulation of calcium pathways in AD that may serve as biomarkers for early diagnosis with therapeutic potential. Calcium-related proteins found in the serum of AD patients have been examined (e.g., TRP, KYNA, 3-HK, QUIN, and PIC) for their role in AD [12]. Notably, 3-HK was found to be upregulated in the serum of AD patients and is known to cross the blood–brain barrier (BBB). It has also been observed that increased degradation of tryptophan via the kynurenine pathway is known to be involved in the molecular mechanisms associated with neuropathogenesis in AD [13,14,15]. QUIN, a metabolite of 3-HK, has been found to localize with hyperphosphorylated tau in the cortical neurons of the brains of AD patients and has been associated with the phosphorylation of tau [16]. Higher QUIN levels in the brains of AD patients have been associated with cognitive impairment, as demonstrated by poor performance on the CAMCOG (the cognitive and self-contained part of the Cambridge Examination for Mental Disorders of the Elderly) test by AD patients [17]. The development of biomarkers for monitoring early events in AD diagnosis represents an unmet medical need.

Alzheimer’s disease (AD) disproportionately impacts racial and ethnic minorities, including African Americans (AAs) and Hispanic/Latinx (H/L) ethnic populations in the United States (U.S.), who are often socioeconomically disadvantaged [18,19,20,21,22]. It has been difficult to explain racial and ethnic disparities in AD; however, it has been proposed that racial disparities may involve vascular pathology observed among AD patients. More specifically, dysregulation of the ET-1 system has been observed in AAs when compared to non-Hispanic Whites (NHWs) [23]. ET-1 is a vasoactive peptide primarily produced by endothelial cells, affecting pericytes and vascular smooth muscle cells [24,25,26]. Pericytes are known to express BQ-123-sensitive ETA receptors found on endothelial cells [27]. It has been observed that African Americans have higher vasoconstriction-mediated blood pressure compared to NHWs in response to stress. Treiber et al. observed higher plasma ET-1 levels in AA males compared to NHW males; AAs also had higher resting plasma ET-1 levels and a greater increase in ET-1 in response to stressors [23]. Higher levels of ET1 are observed in AAs with hypertension compared to normotensive AAs and NHWs [23].

Higher ratios of ET-1 receptors were found in the saphenous veins of hypertensive and normotensive AAs compared to NHWs, which would suggest a racial difference in peripheral vascular resistance and diminished vasodilatation in response to environmental stressors [28]. Palmer et al. demonstrated that ET-1 levels were higher in AD patients and were induced by amyloid beta (Aβ); this was also observed in the postmortem brains of AD patients compared to controls [29]. It has also been shown that ET-1 can induce vasculature constriction under hypoxic conditions via interactions with pericytes [30].

The objective of this study was to obtain information by examining plasma from AD patients and controls to determine if modulation and/or dysregulation of the ET-1 vasoconstrictor is a potential symptomatic contributor to AD pathology. We envision that ET-1 could potentially serve as a blood-based biomarker that could have clinical implications for early diagnosis and novel therapies for the treatment of AD. In addition, we wanted to address the long-standing health disparity in AD among racial and ethnic minorities compared to non-Hispanic whites.

Prior studies suggested that African Americans maintained a higher steady level of ET-1 even in a normotensive state. The sustained high-level ET-1 could potentially have a role in reducing cerebral blood flow, which is an early hallmark feature in AD pathology that could lead to neurodegeneration associated with AD. Unfortunately, we were not able to access specific plasma from a Hispanic/Latinx ethnic cohort, which is a major limitation in this study, along with the small sample size.

## 2. Materials and Methods

The criteria for AD diagnosis and the inclusion criteria of this cohort were provided in the original study by Khan et al. [31]. Plasma was preferred over serum because serum is subject to interference from coagulation or hemolysis, potentially causing errors in biomarker analysis. Plasma offers a richer source of blood components, including blood cells, clotting factors, and various proteins, making it a suitable choice for a wide range of tests. Its versatility allows researchers to carry out proteomics, metabolomics, and coagulation studies with the same sample.

LC/ESI/MS methods can exclusively detect different forms of ET-1, without cross-detection of ET-2 or ET-3, due to the sequence homology of the ET proteins. The plasma protein mixture is separated by using liquid chromatography (LC), then ionizing the peptides generated by enzymatic digestion with electrospray ionization (ESI), and finally analyzing the resulting mass-to-charge ratio (*m*/*z*) on a mass spectrometer, allowing for the identification of distinct protein variants based on subtle mass differences caused by modifications like phosphorylation, glycosylation, or even sequence variations. Standardizations were performed with purchased purified human ET-1.

### 2.1. Preparation of Human Plasma for Sampling

Triethylammonium bicarbonate buffer (TEAB; 1 M), iodoacetamide (IAM), DL-Dithiothreitol (DTT), and sodium dodecyl sulfate (SDS, a detergent) were purchased from Sigma-Aldrich (St. Louis, MO, USA). Human ET-1 was purchased from AnaSpec, Inc. (Freemont, CA, USA). Disulfide bridge 1–15 and 3–11) and ET-1{Leu(13C6,15N), L6}, standard (ET-1_Sdt), with a purity ≥95%, was purchased from GenScript Biotech (Piscataway, NJ, USA). The S-Trap™ micro spin column was purchased from ProtiFi (Farmingdale, NY, USA). Endothelin has a high affinity for bonding with albumin in human plasma and is an intra-membrane hydrophobic protein (polypeptide). The protein (1.60 μL of plasma from patients, which represents ~ 60 μg to 80 μg of protein) was solubilized with 40 µL of 5% SDS in 50 mM of ammonium bicarbonate. The sample was spiked with 50 fmol of ET-1_Sdt and reduced for 10 min at 56 °C with DTT (final concentration at 20 mM), followed by acetylation with IAM (final concentration at 40 mM), for 30 min with incubation at room temperature in the dark, which blocks all cysteines and adds *m*/*z* 54. The proteins were acidified with a final concentration of 1.2% phosphoric acid and diluted with six volumes of S-Trap™ buffer (90% Methanol, 100 mM TEAB, pH 7.1) to aggregate proteins in colloidal particles. The samples were transferred into S-Trap™ columns, trapped in a filter by one centrifugation at 4000× *g* for 2 min, and washed four times with 150 μL of S-Trap™ buffer to remove SDS. Peptide elution was conducted using (i) 40 μL TEAB 50 mM; (ii) 0.2% formic acid (HCOOH) in H_2_O; and (iii) 35 μL of 50% acetonitrile (CH_3_CN), with a final concentration of 0.2% formic acid. Centrifugations were performed at 4000× *g* for 2 min between each elution buffer. Eluates were pooled, lyophilized in a speed vacuum, and re-suspended in 20 μL of 95:5 (H_2_O/CH_3_CN) and 0.2% HCOOH. The sample injection volume was 5 μL.

### 2.2. Liquid Chromatography–Tandem Mass Spectrometry

Peptides were identified using an Ultimate Waters™ LC system (Waters Corporation, Milford, MA, USA) with an autosampler injector, coupled to the Thermo Scientific™ TSQ Vantage Triple Stage Quadrupole Mass Spectrometer (Thermo Fisher Scientific, Waltham, MA, USA). Nitrogen was used for both sheath and auxiliary gases, with pressures set at 10 and 5 (arbitrary units), respectively. The instrument operated in a positive ion mode [32]. The flow electrospray needle voltage and declustering voltage were set at 4000 V and 10 V, respectively. Capillary temperature was set at 300° C. Collision pressure was set at 1.0 mTorr. For all reactions, the scan width for product ions was set at 0.5 (*m*/*z*), and the cycle time for each ion was set at 0.5 s. The peak width for Q1 and Q3 was set at 0.7 (full width at half maximum). Data acquisition was set to profile mode.

**Figure 1:**
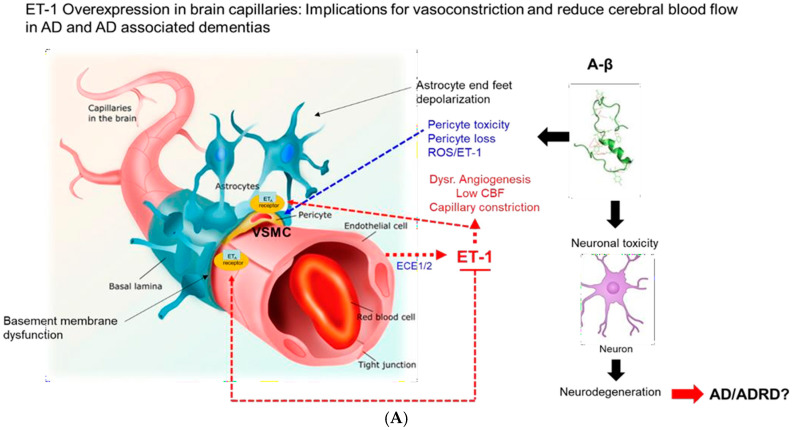

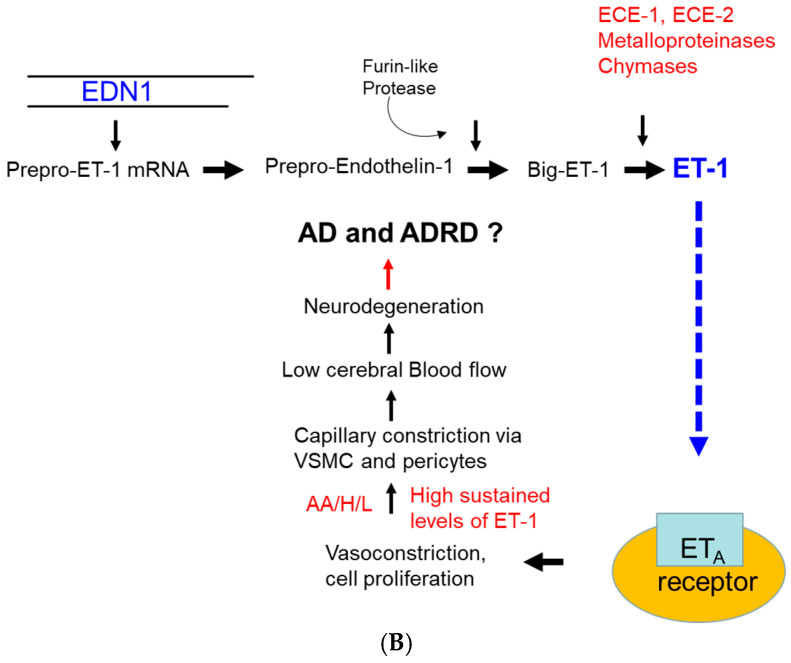
(**A**). ET-1 overexpression in brain capillaries: Implications for vasoconstriction and reduced cerebral blood flow in AD and ADRD. (**B**). The predicted role for ET-1 in AD. The expression of endothelins (ET-1, ET-2, and ET-3) occurs on separate mRNA transcripts and is encoded by END-1, EDN-2, and EDN-3 gene loci. The resulting preproendothelin mRNAs are translated to form their respective preproendothelins 1–3. The endothelins are proteolytically cleaved by furin-like proteases from the respective Big ETs (Big ET-1, Big ET-2, and Big ET-3). Metalloproteinases and chymases, produce the active peptide forms, respectively. Active forms of endothelins bind to G-protein receptors ETA and ETB to activate cellular functions via ETB receptor binding by ET-1–3, resulting in vasodilation and inhibition of growth and inflammation; ET-1 and ET-2 may activate the cellular function by binding to ETA receptors on vascular smooth muscle cells (VSMCs) and pericytes in the brain and induce vasoconstriction and cell proliferative responses. Figure 1 was partially derived from a figure at https://pmc.ncbi.nlm.nih.gov/articles/PMC7712547/ accessed on 13 January 2025.

**Figure 2:**
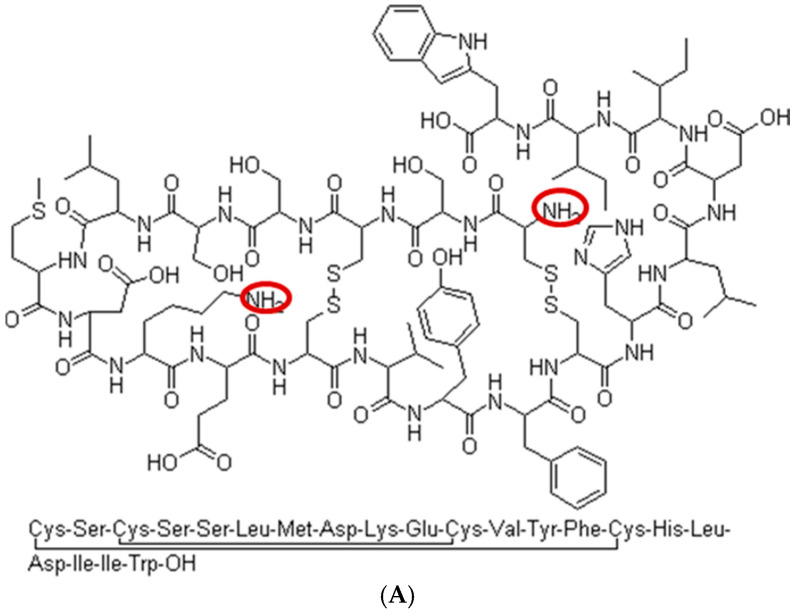

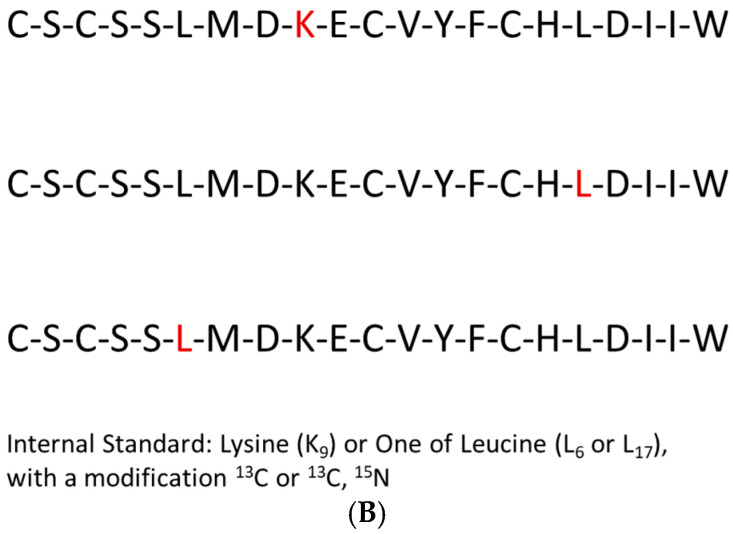
(**A**)The amino acid sequence and structure of ET-1. (**B**) Labeling of the ET-1 standards.

The temperature settings for the column and sample were set, respectively, to 40 °C and 4 °C. Peptides were analyzed using selected reaction monitoring (SRM) on Acquity UPLC BEH C18 column (Waters Corporation), 1.7 µm (2.1mm × 50 mm, at a flow rate of 0.3 μL/min^−1^ using an 11-min gradient of mobile phase A [0.1% HCOOH/100% H2O] and phase B [0.1% HCOOH/100% CH3CN]) [33,34]. The gradient used was 15% B for 0 min to 2.5 min; 2.5% to 7% B from 15% to 99%; and 7 min to 7.5 min at 99% B, then returned to 15% for equilibration, from 7.5 min to 11 min. Full MS scans were acquired from 100 *m*/*z* to 1500 *m*/*z*, and only ions with two and three charges were selected for MS/MS analysis. In ET-1, all four cysteines were alkylated (the total mass added equaled 228.16). Using an SRM program, the selected reactions from the triply protonated molecule *m*/*z* 909 [M + 3H]^3+^ demonstrated highly selective cleavage toward the double-charged fragment b^2+^20 (*m*/*z* 1261), representing the elimination of the terminal tryptophan amino acid. Other fragments include b^2+^19 (*m*/*z* 1204), b^2+^18 (*m*/*z* 1148), and b^2+^17 (*m*/*z* 1091), and triple-charged fragments b^3+^20 (*m*/*z* 840), b^3+^19 (*m*/*z* 803), and y^3+^14 –H2O (*m*/*z* 765). *m*/*z* 318 is the tryptophan fragment. Respectively, for the standard (*m*/*z* 911) [M + 3H]^3+^, we have the following fragments: double-charged fragments b^2+^20 (*m*/*z* 1264); b^2+^19 (*m*/*z* 1207); b^2+^18 (*m*/*z* 1151); and b^2+^17 (*m*/*z* 1091), and triple-charged b^3+^20 (*m*/*z* 843); b^3+^19 (*m*/*z* 805); and y^3+^14 –H2O (*m*/*z* 767). *m*/*z* 318 is also the tryptophan fragment. The fragments and chromatograms are shown in Figure 1 and Figure 2.

### 2.3. Endothelin Peptide Quantification

**Figure 3:**
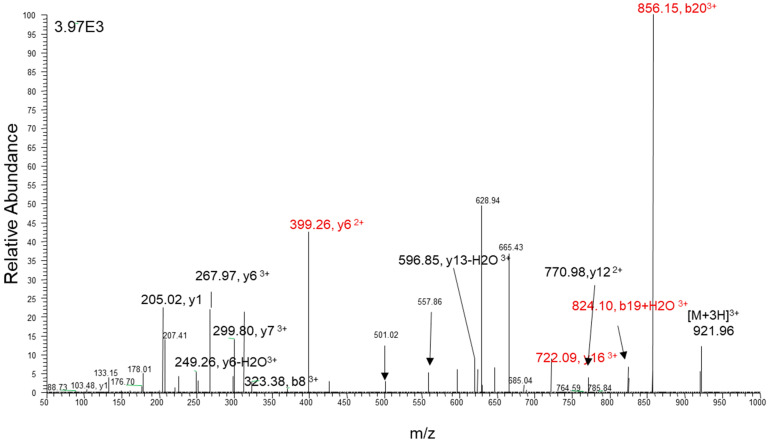
The LC/ESI/MS spectra and selected ion monitoring of ET-1 derivative (PITC-ET-1 *m*/*z* 921.96, [M + 3H]^3+^) are shown. The spectra recorded at −28 eV contain all major fragments generated by CID. The product ions were scanned from *m*/*z* 50 to *m*/*z* 1000. The fragment ions at *m*/*z* = 856 (b20 ^3^+) and 399 (y6^3+^) were used for quantification (*m*/*z* 824 (b19+H_2_O^3+^) and 722 (y16^3+^) as qualifiers.

**Figure 4:**
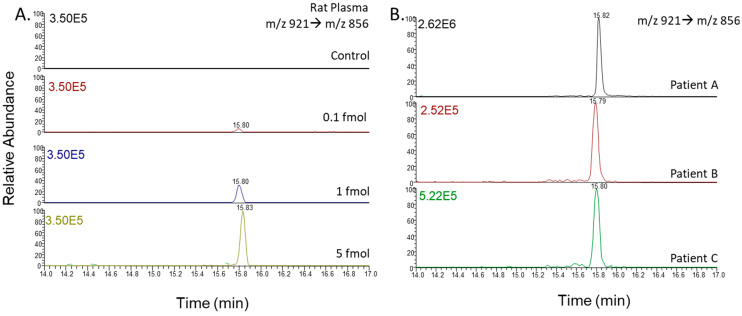
PITC-ET-1 in blank rat plasma or human plasma; (**A**) 10 µL of rat plasma spiked at 0.1, 1, and 5 fmol; (**B**) 10 µL patient sample (A: lot PL101614A, B: lot 012815D, C: PL020520C) were analyzed via LC/ESI/MS/MS. The selected reaction monitoring mode was used to carry out the quantitative analysis; the specific transition ions for the precursor ions at *m*/*z* 921→*m*/*z* 856 are shown.

As shown in Figure 3 and Figure 4, ET-1 has a highly selective cleavage toward *m*/*z* 1261 (the b^2+^20 ion), which represents the elimination of the terminal tryptophan amino acid (*m*/*z* 318). Identical shifts and collision energies were observed for the standard ET-1_Sdt [^13^C, ^15^N] at *m*/*z* 1264. Label-free quantification of proteins is based on the area under the curve; the signal intensity of the peptide (ET-1) is divided by the area under the curve of the peptide signal intensity of the internal standard (ET-1_Sdt) times the amount in fmol of the standard injected onto the column. The amount for each sample is expressed in fmol. An expression in μg by μL (ng/mL) is multiplied by MW of ET-1 (MW = 2491.9) and then divided by 1.60 μL (volume used).

### 2.4. Data Curation

Two datasets were retrieved from the Vanderbilt University Medical Center Proteomics Core (Nashville, TN, USA) [19]. The mean of two ET-1 replicates was used in the analysis as a measure of plasma concentration in ET-1. Both datasets were merged, based on the shipping ID, to create a dataset with a total of 113 observations. The Alzheimer’s classifications of patients were grouped into probable AD (probable AD, probable AD w/atypical presentation, probable AD w/atypical presentation w/subcortical white matter lucencies, or probable AD w/subcortical white matter lucencies) and control (CAMCI control, control, control w/subcortical white matter lucencies, control with cerebrovascular disease, control with head injury, family genetic study control, Lewy body variant dementia, movie control, or not demented [subjective complaint with normal test scores] w/SWML) groups. Differences in age at draw and mean ET-1 score, stratified by race, sex, and AD status, were conducted using an independent *t*-test. Associations among pairs of categorical explanatory variables were calculated. Spearman, biserial, and partial correlations were used to measure the desired associations. Lastly, a multivariable logistic regression was used to measure the effect of ET-1 on AD, controlling for sex, race, and age at draw. The odds ratios and their corresponding 95% confidence intervals were calculated. IBM SPSS version 28.0.0.0 (190) was used to conduct all analyses.

## 3. Results

Descriptive analysis in Table 1 shows that the mean age at draw was 73.65, with a standard deviation of 8.45 years, and the mean concentration of ET-1 was 39.10 ng/mL, with a standard deviation of 22.78 ng/mL. Stratifying age at draw based on sex revealed that males were slightly older (75.12 years old) than females (72.94 years old), and those with probable AD were slightly older (75.45 years old) than patients without AD (71.75 years old), as seen in Table 2. Although mean ET-1 scores were lower in females, NHWs, and those with probable AD compared with males, AAs, and those without probable AD, respectively, these differences were not statistically significant. Mean and standard deviations were calculated for subjects within the AD status groups, stratified by race, as seen in Table 3.

Conditional probabilities (Table 4) revealed that the vast majority of AAs (71.4%), NHWs (63.2%), those with probable AD (67.2%), and those without probable AD (67.3%) were females.

The Spearman correlation showed a significant correlation between age at draw and ET-1 (r = 0.186, *p* = 0.048). For AAs, the Spearman correlation between age at draw and ET-1 was 0.369 (*p* = 0.005), while for NHWs, the correlation was not significant (r = 0.052, *p* = 0.702). The correlation values between age at draw and ET-1 for males and females were −0.112 (*p* = 0.508) and 0.308 (*p* = 0.007), respectively. No significant difference was found between the mean ET-1 for males and females (*p* = 0.237). No significant difference was found between the male and female mean age at draw (*p* = 0.199).

**Figure 5:**
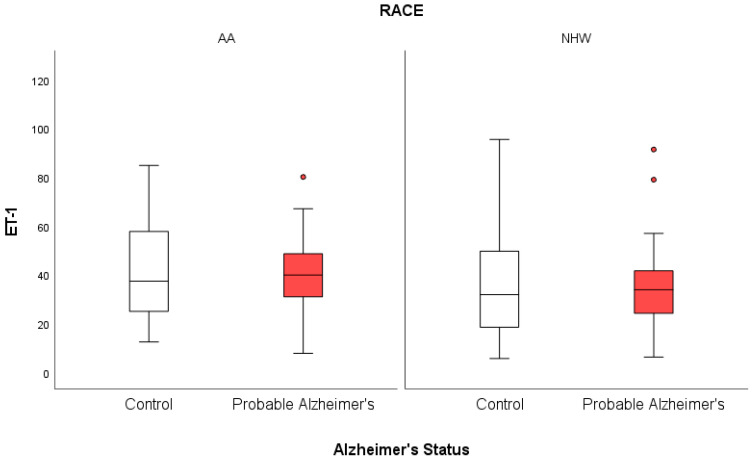
The distribution of ET-1 scores based on AD status and racial group is displayed in the form of a boxplot. The bottom and top of each box reflect the 25th and 75th percentiles, respectively, with the line in the middle of the box representing the 50th percentile (median).

When stratifying by race and AD status, AAs with probable AD were found to be the only group whose correlation between age at draw and ET-1 was significant (r = 0.369, *p* = 0.049), as seen in Figure 5. The correlation between age at draw and ET-1 was not significant for AAs without probable AD (r = 0.308, *p* = 0.118), NHWs with probable AD (r = −0.035, *p* = 0.857), and NHWs without probable AD (r = 0.117, *p* = 0.555). Stratifying by sex and AD status revealed a significant correlation between age at draw and ET-1 (r = 0.343, *p* = 0.038), but not for females with probable AD (r = 0.256, *p* = 0.115), males without probable AD (r = −0.117, *p* = 0.645), or males with probable AD (r = −0.263, *p* = 0.276). Spearman point-biserial correlation between ET-1 and AD status among AAs was 0.072 (*p* = 0.599); among NHWs, it was –0.023 (*p* = 0.971). No significant point-biserial correlation was found between ET-1 and AD status among females (r = 0.013, *p* = 0.914) or males (r = 0.035, *p* = 0.835). No significant difference in mean ET-1 score was found between non-AD and AD groups, whether within AA (*p* = 0.929) or NHW (*p* = 0.388) patient populations.

For AAs, the partial correlation between age at draw and ET-1, controlling for AD status, was 0.314, with a *p*-value of 0.019. For NHWs, the partial correlation was −0.023, with a *p*-value of 0.862.

**Figure 6:**
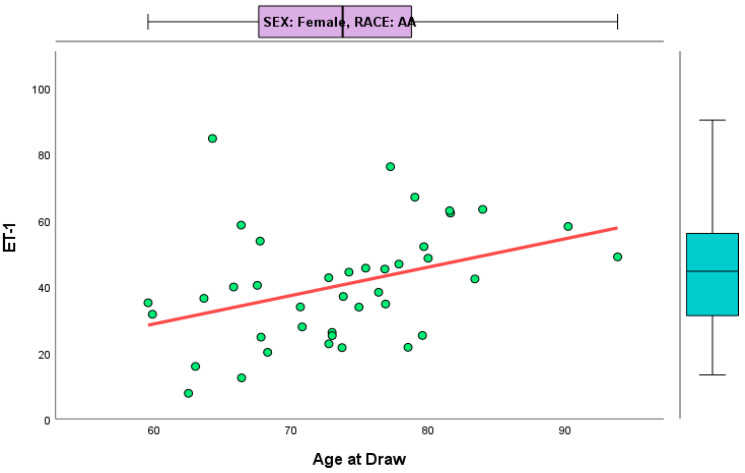
This scatterplot illustrates the relationship between age at draw and ET-1 scores for AA females. The linear relationship, as represented by the red line, reveals a positive association between the two variables. Box plots on the right and top of the figure show the distribution of ET-1 and age at draw variables.

Correlations were calculated between age at draw and ET-1, stratified by both race and sex. The only significant correlation belonged to female AAs (r = 0.465, *p* = 0.002). Figure 6 shows the linear association between age at draw and ET-1 for AA females. Female NHWs (r = 0.143 *p* = 0.407), male AAs (r = 0.138, *p* = 0.610), and male NHWs (r = −0.242, *p* = 0.291) were not found to be significant.

The Spearman correlation of age at draw and ET-1, stratified by race and sex, while controlling for AD status, was significant for female AAs (r = 0.385, *p* = 0.016). Partial correlation was not significant between age at draw and ET-1, when controlling for AD status.

As expected, multivariable logistic regression results (see Table 5) showed that age at draw was significant in predicting AD (*p* = 0.017) when controlling for race, sex, and ET-1. The adjusted odds ratio (AOR) for AAs with probable AD was 8.3% higher than for NHWs (AOR = 1.083, *p* = 0.839). Females had a 7.2% higher AOR of probable AD than males (AOR = 1.072, *p* = 0.869). The AOR for an individual with probable Alzheimer’s was 0.991 (*p* = 0.319). For each year’s increase in age at draw, there was a 6% increase in the odds of an individual having probable AD (AOR = 1.060, *p* = 0.017).

## 4. Discussion

Based on our data, ET-1 was clearly not a significant factor in predicting AD. However, a significant correlation was discovered between age at draw and ET-1 in AA women. The limitations of this study include the small number of specimens involved, the absence of specimens from H/L individuals with and without AD, and the need for validation of these studies with correlative information on Aβ and Tau deposition. This study was designed to determine if ET-1 levels are higher in AD patients compared to controls; if so, then this would be the basis for determining if this vasoconstrictor would play a role in AD development as well as help to explain the racial disparity seen in African Americans with AD compared to non-Hispanic White patients with AD. This is a long-standing disparity that will require further investigation.

There is also a disparity among Hispanic/Latinx ethnic populations with AD that is unexplained and will require strategies to include more Hispanic AD patients in clinical trials. Disparity in AD clinical trial access will lead to disparities in access to innovative therapies for AD. The concept of this study could be used for the elucidation of new approaches to symptomatic treatments for AD. AD is multifactorial and understanding the symptomatic contributions of this complex disease could provide information for the development of a stepwise approach to limit disease progression, and/or a combinatorial strategy that could lead to early diagnosis and potential curative therapies. Understanding the role of host cell factors like ET-1 in AD development is essential to determining the pathological mechanisms of AD.

Moreover, novel strategies, such as non-invasive brain stimulation (NIBS), have been shown to improve cognitive and neuropsychiatric symptoms, which can improve the quality of life of AD patients [34,35,36]. Brain stimulation has been shown to modulate cognitive functions in neuropsychiatric diseases. NIBS include transcranial magnetic stimulation (TMS), cranial electrostimulation (CES), transcranial alternating current stimulation (tACS), electroconvulsive treatment (ECT), transcranial direct current stimulation (tDCS), magnetic seizure therapy (MST), and non-invasive VNS [37,38,39,40,41,42,43]. Although NIBS has shown promise and potential as an innovative intervention for the treatment of AD, studies using NIBS for AD have shown inconsistent results from studies that were often poorly designed with regard to patient selection, population, and sample sizes [44]. A comprehensive meta-analysis of 19 different studies showed that NIBS significantly improved global cognition and neuropsychiatric symptoms (NPSs) in AD. MCI and repetitive transcranial magnetic stimulation (rTMS) significantly improved global cognition and NPS in AD [44]. Most recently, transcranial pulse stimulation (TPS) has been shown to improve depression in AD patients [45]. Matt et al. showed that stimulating areas related to depression, including the extended dorsolateral prefrontal cortex, appears to alleviate depressive symptoms and induce functional connectivity (FC) changes in AD patients [45]. FC analysis showed a normalization of the FC between the salience network (right anterior insula) and the ventromedial network [45]. There is great interest in further developing NIBS as an innovative therapy for the treatment of AD but this will require more standardized study designs with larger sample sizes and long-term follow-up.

## 5. Limitations and Future Directions

The sample size in this study is an important limitation that needs to be addressed in AD research overall. The small sample size may reflect the limited inclusion and/or participation of African American and Hispanic/Latinx ethnic populations in AD clinical trials [46,47]. There were no specimens from Hispanic/Latinx ethnic AD patients in this study. Hispanic Latinx populations in the US are projected to experience the greatest increase in AD in the next 40 years, compared to other major ethnic groups. Hispanic/Latinx communities are at an increased risk for AD and other dementias, with limited access to quality care [48]. The ET-1 assay was developed and standardized empirically in-house and it was not a routine commercial assay. The amount of plasma from each patient was limited and would not allow for a comparative analysis in different assays. Larger and more diverse studies are necessary to validate and further explore these findings for the early detection of minor cognitive disorders. We are also aware that different subtypes of Alzheimer’s disease (AD) might have varying effects on ET-1 plasma levels; moreover, the potential variability in the control group could also influence ET-1 levels. Future studies will need to focus on increasing the sample sizes of cohorts by coordinating with larger ongoing studies and encouraging the recruitment of minorities in AD research and clinical trials. Moreover, efforts should be made to include a significant sample size that allows for the examination of plasma specimens from different AD subtypes.

## 6. Conclusions

To thoroughly examine the data, we conducted different analyses based on our hypotheses when performing these tests. Because multiple comparisons were not used, there was no need to adjust the type I error probability. The statistical power was not affected as a result of conducting these different statistical tests. We examined different aspects of the data, which required different types of tests. Initially, we compared variables directly and subsequently controlled for potential confounders. For example, when using the *t*-test, we compared the means of two continuous variables. Correlation methods allowed us to measure the degree of their association. For instance, biserial correlation was used to measure the degree of association between the binary variable (AD) and continuous variable (ET1). Later, we used multivariable logistic regression to test the same effect of ET1 on AD, while controlling for other potential confounders. Based on our data, ET1 was not a significant predictor of Alzheimer’s disease.

It has been approximately 30 years since the passage of the 1993 National Institute of Health Revitalization Act, which required the inclusion of women and racial/ethnic minority groups into government-funded clinical trials. Minority groups have remained under-represented, which has contributed to existing health disparities [49]. The key to advancing equitable research in AD will be to develop a systematic action plan to recruit AD patients, individuals at high risk for AD, and family members of AD patients to participate in clinical trials focused on AD and ADRD. This strategic plan must start at the level of AD education, awareness, and training, which would require certification for the debunking of myths surrounding AD, clinical trials, and mistrust of the medical providers. This plan would also require community healthcare workers to serve as liaisons, especially in underserved communities that require AD health-related services. This would enhance the level of awareness and education required to increase the participation of ethnic minorities in AD research and clinical trials, which is essential for addressing health disparities in AD. This would provide opportunities for advancements in AD research, supporting the goals of developing equitable innovation in therapeutics that could impact AD treatment and AD-related health disparities. The dysregulation of ET-1 signaling pathways at different stages of AD could reveal important changes in early-stage cognitive decline, but these changes may not show significant differences in late-stage AD. Additional studies that could further elucidate ET-1 relevance as a biomarker should include larger sampling sizes of plasma specimens from early and late-stage AD; this could provide insights that might remain unresolved in smaller studies. The link between ET-1 and vascular health and AD has been reported. The link between ET-1 and hypertension has also been reported (hypertension is a known risk factor for AD). Sustained hypertension over time is associated with low cerebral blood flow, which can contribute to neuronal damage, which is an associated precursor to dementia (Figure 1).

There are many challenges to studying AD biomarkers in diverse populations. Underserved communities may lack infrastructure support to conduct AD research locally and may require participants to travel long distances and incur financial hardships to participate in clinical research. The longstanding distrust of the government and medical establishment among diverse communities has made the sustained recruitment of diverse populations difficult. Strategies to address infrastructure support and mistrust regarding AD research could include funding Historically Black Colleges and Universities (HBCUs) and Hispanic-serving institutions (HSIs) to conduct AD biomarker research in these diverse populations. This strategy could improve minority participation rates in AD biomarker research, support health equity in AD, and reduce AD-associated health disparities.

Correlations between ET-1 and age in AA women are unclear. However, African American women are at a high risk of developing dementias, such as AD, and face disproportionate social and environmental pressures as well as healthcare prejudice, leading to barriers that can restrict access to AD care. Studies show that sex and sex hormones can affect ET-1 levels and ETA and ETB receptors [50]. Aging can lead to dysregulation of the ET-1 system, resulting in an imbalance between vasodilation and vasoconstriction. Therefore, ET-1 could play a role in the sex differences observed with vascular aging. In addition, menopause transition is associated with an accelerated risk of vascular dysfunction, including increased cardiovascular disease risk [51] The sex-specific decline in endothelial function observed in women after menopause is greater than in men as they age, and is greatly accelerated [52]. This process can contribute to ET-1 system dysfunction and vascular disease that could predispose these women over time to the acquisition of AD. This process may be enhanced in African American women as they age and could be attributable to genetics, lifestyle factors, and/or environmental exposures.

## Figures and Tables

**Table 1:** Descriptive statistics for overall age at draw and ET-1, including the mean, standard deviation, and 95% confidence intervals around the mean, median, minimum, and maximum values.

Variable Name	Mean (±SD)	95% CI	Median	Min	Max
**Age at draw**	73.65 (±8.45)	(72.08, 75.23)	73.80	53.07	93.85
**ET-1**	39.10 (±22.78)	(34.85, 43.35)	34.70	5.58	120.19

**Table 2:** Descriptive statistics of the continuous variables for age at draw and ET-1, stratified by sex, race, and AD. Mean and standard deviations, along with their corresponding 95% confidence intervals, as well as the median, minimum, and maximum values, are reported.

Variable	Stratification	Mean (±SD)	95% CI	Median	Min	Max
**Age at draw**	Male	75.12 (±7.96)	(72.47, 77.78)	76.83	59.96	86.78
	Female	72.94 (±8.64)	(70.96, 74.91)	73.75	53.07	93.85
	AA	73.57 (±7.66)	(71.52, 75.63)	73.35	59.54	93.85
	NHW	73.73 (±9.22)	(71.28, 76.18)	74.51	53.07	89.26
	Probable Alzheimer’s	75.45 (±8.33)	(73.26, 77.65)	77.31	56.38	93.85
	No Probable Alzheimer’s	71.75 (±8.22)	(69.53, 73.98)	71.32	53.07	90.23
**ET-1**	Male	42.74 (±26.16)	(34.02, 51.47)	34.67	9.58	120.19
	Female	37.33 (±20.90)	(32.55, 42.10)	34.88	5.58	104.94
	AA	41.07 (±18.75)	(36.05, 46.10)	37.77	7.71	84.78
	NHW	37.16 (±26.17)	(30.22, 44.11)	33.44	5.58	120.19
	Probable Alzheimer’s	37.51 (±17.91)	(32.81, 42.22)	35.05	6.16	91.31
	No Probable Alzheimer’s	40.78 (±27.06)	(33.46, 48.09)	33.47	5.58	120.19

**Table 3:** Stratifying data based on race and AD status; the mean and standard deviations for ET-1 are displayed.

African American Control	Mean 41.307	Standard Deviation 21.904
African American with probable Alzheimer’s	40.856	15.662
Non-Hispanic White control	40.266	31.656
Non-Hispanic White with probable Alzheimer’s	34.166	19.608

**Table 4:** M = Male; F = female; AA = African American/Black; NHW = non-Hispanic White; ALZ = probable AD; No ALZ = no probable AD. Percentages listed in the table are conditioned on column label grouping and are shaded according to percentage (light brown: 0 –< 30%|darker brown: 30 –< 60%|darkest green: 60+%).

	M	F	AA	NHW	ALZ	No ALZ
M			28.6	36.8	32.8	32.7
F			71.4	63.2	67.2	67.3
AA	43.2	52.6			50.0	49.1
NHW	56.8	47.4			50.0	50.9
ALZ	51.4	51.3	51.8	50.9		
No ALZ	48.6	48.7	48.2	49.1		

**Table 5:** AD status regressed on race, sex, ET-1, and age at draw variables. Multivariable logistic regression results consist of the beta coefficients, standard errors, Wald test statistics, *p*-values, adjusted odds ratio, and respective 95% confidence intervals for each dependent variable.

	Coefficient	S.E.	Wald	df	*p*-Value	Adjusted Odds Ratio	95% CI
**Intercept Race**	−3.954	1.857	4.532	1	0.033	0.019	
African American	0.079	0.392	0.041	1	0.839	1.083	(0.503, 2.332)
Non-Hispanic White	ref					1	
**Sex**							
Female	0.069	0.421	0.027	1	0.869	1.072	(0.470, 2.446)
Male	ref					1	
**ET-1**	−0.009	0.009	0.992	1	0.319	0.991	(0.974, 1.009)
**Age at draw**	0.058	0.024	5.657	1	0.017	1.060	(1.010, 1.112)

Variable(s): race, sex, ET-1, age at draw. Outcome.

## Data Availability

The datasets used and/or analyzed during the current study are available from the corresponding author upon reasonable request.
